# *TRT*, a Vertebrate and Protozoan *Tc1*-Like Transposon: Current Activity and Horizontal Transfer

**DOI:** 10.1093/gbe/evw213

**Published:** 2016-09-25

**Authors:** Hua-Hao Zhang, Guo-Yin Li, Xiao-Min Xiong, Min-Jin Han, Xiao-Gu Zhang, Fang-Yin Dai

**Affiliations:** ^1^ State Key Laboratory of Silkworm Genome Biology, Key Laboratory for Sericulture Functional Genomics and Biotechnology of Agricultural Ministry, Southwest University, Chongqing, China; ^2^ College of Pharmacy and Life Science, Jiujiang University, Jiujiang, China; ^3^ Department of pathology, Hanzhoung Hospital, Hanzhoung city, Shaanxi, China; ^4^ Clinical Medical College, Jiujiang University, Jiujiang, China

**Keywords:** Tc1/mariner transposons, DD37E, horizontal transfer

## Abstract

We report a *Danio rerio* transposon named *DrTRT*, for *D. rerio* Transposon Related to *Tc1*. The complete sequence of the *DrTRT* transposon is 1,563 base pairs (bp) in length, and its transposase putatively encodes a 338-amino acid protein that harbors a DD37E motif in its catalytic domain. We present evidence based on searches of publicly available genomes that *TRT* elements commonly occur in vertebrates and protozoa. Phylogenetic and functional domain comparisons confirm that *TRT* constitutes a new subfamily within the *Tc1* family. Hallmark features of having no premature termination codons within the transposase, the presence of all expected functional domains, and its occurrence in the bony fish transcriptome suggest that *TRT* might have current or recent activity in these species. Further analysis showed that the activity of *TRT* elements in these species might have arisen about between 4 and 19 Ma. Interestingly, our results also implied that the widespread distribution of *TRT* among fishes, frog, and snakes is the result of multiple independent HT events, probably from bony fishes to snakes or frog. Finally, the mechanisms underlying horizontal transfer of *TRT* elements are discussed.

## Introduction

Transposable elements (TEs) are DNA fragments that move from one host genomic site to another. TEs have been reported in almost all organisms, including plants, invertebrates, vertebrates, fungi, and bacteria, and they occupy a substantial fraction of various host genomes (Biémont 2010). TEs that integrate into a new genomic location play important roles in genome architecture as well as in genetic innovation (Feschotte and Pritham 2007).

TEs are generally classified into two classes (Classes I and II) based on their structural organization and mechanism of transposition (Feschotte and Pritham 2007). Class I or RNA elements are transposed via reverse transcription of an RNA intermediate, whereas that Class II or DNA elements transpose directly as DNA, mostly through a so-called “cut and paste” mechanism. The *Tc1/mariner* superfamily of TEs is the most widespread class of DNA transposons in nature (Feschotte and Pritham 2007). *Tc1/mariner*, which was first discovered in *Drosophila mauritiana* (Haymer and Marsh 1986; Jacobson et al. 1986), is ubiquitous in eukaryotes (Feschotte and Pritham 2007; Liu and Yang 2014). *Tc1/mariner* elements are generally 1,300–2,400 bp in size and encode a 340-amino acid transposase that is flanked by terminal inverted repeats (TIRs) and a target site duplication (TSD), TA (Lohe et al. 1996). On the basis of variations in the DDE/D signature motif, *Tc1/mariner* elements are further classified into various monophyletic groups (Shao and Tu 2001). Although some naturally active *Tc1/mariner* elements have been identified in nematodes and arthropods, most of these are inactive in vertebrates due to the occurrence of stop codons, deletions, or frameshifts. Importantly, active *Tc1/mariner* transposons may be potentially used as molecular tools for transgenesis and insertion mutagenesis (Pavlopoulos et al. 2007; Voigt et al. 2016). To date, only some *Tc1*-like elements (such as *Tol1*, *Tol2*, *Passport*, and *Tana1*) in bony fishes are known to be both intact in their native form and transpositionally active (Koga et al. 2006; Clark et al. 2009; Pujolar et al. 2013; Watanabe et al. 2014).

TEs are ubiquitous in organisms despite extensive evidence that their presence and mobility causes a variety of deleterious mutations (Craig et al. 2002). One explanation is that TEs have the ability to invade a new host by horizontal transfer (HT). A previous study demonstrated that almost all kinds of eukaryotic TEs are capable of HT (Schaack et al. 2010). *Tc1/mariner* elements are highly capable of invading a wide range of species because these are not dependent on host factors to mediate their mobility. Although more than 94 HT cases of *Tc1/mariner* elements have been reported, these phenomena mainly occur between or among invertebrates (Dotto et al. 2015). HT events occurring in vertebrates mainly involve the *hAT* superfamily of DNA transposons (Pace et al. 2008; Novick et al. 2010; Gilbert et al. 2010; Gilbert et al. 2012; Gilbert et al. 2013).

In the present study, we performed a detail analysis of a *Tc1*-like transposon in the zebrafish (*Danio rario*). Further analysis confirmed that *TRT* elements constitute a new subfamily within the *Tc1* family. We also show that *TRT* elements are both intact in their native form and functionally active in *Pundamilian yererei*, *Maylandia zebra*, and *Haplochromis burtoni*. Finally, we demonstrate that *TRT* elements might have invaded into its hosts via multiple HT events.

## Materials and Methods

### Identification and Copy Number Determination of *TRT*

TBLASTN analysis (Altschul et al. 1990) of the zebrafish genome was performed using the amino acid sequences of *Bombyx mori Bmmar1* (U47917) as query, which in turn detected a *Tc1/mariner* transposon that encoded a DD37E motif-harboring polypeptide. We named these elements *DrTRT* (an abbreviation for *Danio rerio* Transposon Related to *Tc1*) because of its several similarities to transposons of the *Tc1* family, as described in Results part. To determine the distribution of *DrTRT*, its transposase sequence was used as query to search against 1,176 species genomes, including 437 fungus, 216 invertebrates, 226 vertebrates, 150 plants, and 147 protozoans that are available at National Center for Biotechnology Information (NCBI). This transposon was determined to exist in one species when the unique DD37E motif was detected in the catalytic domain of one transposon.

The whole-genome shotgun sequences of all studied species were downloaded from NCBI. The consensus sequences of *TRT* were reconstructed using a multiple alignment of full-length copies in each genome using DAMBE (Xia and Xie 2001). Then, these respective consensus sequences were employed to mask each host genome to estimate copy number. All BLAST hits with more than 100 bp in size and 80% identity were used to calculate copy number.

### Sequence and Phylogenetic Analyses

Inverted repeats were manually searched by the FastPCR software (Kalendar et al. 2014). The potential open reading frame of *TRT* used in the present study was analyzed using GENSCAN (http://genes.mit.edu/GENSCAN.html) or getorf in the EMBOSS-6.3.1 package (Rice et al. 2000), with default parameters. A multiple alignment of these elements was created by MUSCLE (Edgar 2004), with default parameters. The secondary structure of transposase was predicted by PSIPRED (McGuffin et al. 2000), with default parameters. Putative nucleus localization signal (NLS) motifs were predicted using PSORT II Prediction as provided in the PSORT WWW server (http://psort.nibb.ac.jp/). Each pairwise identity was calculated by Bioedit (Hall 1999) after all ambiguous and gapped sites were removed.

Sequences of Recombination-activating gene 1 (*RAG1*) were used in the comparison with transposon distance, with the purpose of testing HT hypothesis. Their accession numbers were listed in supplementary table S4, Supplementary Material Online. Multiple alignments of *RAG1* and *TRT* were created using MUSCLE (Edgar 2004). Then, comparison distances of *RAG1* and *TRT* were calculated using MEGA 4 (Tamura et al. 2007; pairwise deletion, maximum composite likelihood) based on two aligned files (supplementary dataset S1 and S2, Supplementary Material Online).

Transposase sequences of *Tc1*, *mariner*, and *maT* were downloaded from GenBank. The accession numbers are as follows: *Anopheles albimanus Qeutzal* (L76231), *Pleuronectes platessa PplTc1* (AJ303069), *Haemonchus contortus HcTc1* (AF099908), *Rana pipiens RpiTc1* (BK001476), *Drosophila virilis Paris* (Z49253), *Fusarium oxysporum Impala* (AF282722), *Ceratitis capitata Ccmar1* (U40493), *Homo sapiens Hsmar1* (U52077), *H. sapiens Hsmar2* (U49974), *Drosophila mauritiana Dmmar1* (X78906), *Drosophila simulans Dsmar1* (X89927), *Chrysoperla plorabunda Cpmar1* (U11650), *Girardia tigrina Dtmar1* (X71979), *Adineta vaga Avmar1* (DQ138246), *Apis mellifera Ammar1* (AY155490), *Droshophila mauritiana Mos1* (AEZ51500), *Forficula auricularia Famar1* (AAP51098), *Shigella sonnei IS630* (X05955), *Bombyx mori Bmmar1* (U47917), *B. mori Bmmar6* (AF461149), *Caenorhabditis briggsae CbmaT1* (AC084526); *C. briggsae CbmaT4* (AC084524), *C. briggsae CbmaT5* (AC084578), *C. elegans CemaT1* (U41268), and *Philodina roseola PrD37E* (DQ138288). *PrD37D* (*P. roseola*), *Tc1* (*C. elegans*), *Tc1-1_Pm* (*Petromyzon marinus*), *Tc1-1_Lch* (*Latimeria chalumnae*), *Tc1-1_Dr* (*D. rerio*), and *Tc1-1Ory* (*Oryzias latipes*) were downloaded from Repbase (Jurka et al. 2005). A multiple alignment of full-length transposase sequences of the above transposons and all *TRT* was created by MUSCLE with default parameters (Edgar 2004). Then, the appropriate amino acid (aa) substitution model was selected using the Akaike information criterion in the ProtTest 3 server (Darriba et al. 2011). The best-suited aa substitution model for these data was the WAG model. Phylogenetic trees were then built using the MRBAYES 3.1.2 software (Ronquist and Huelsenbeck 2003) until the values of the average SD of split frequencies were stably below 0.01.

## Results

### Identification and Characteristics of *DrTRT* in the Zebrafish Genome

**Fig. 1.—:**
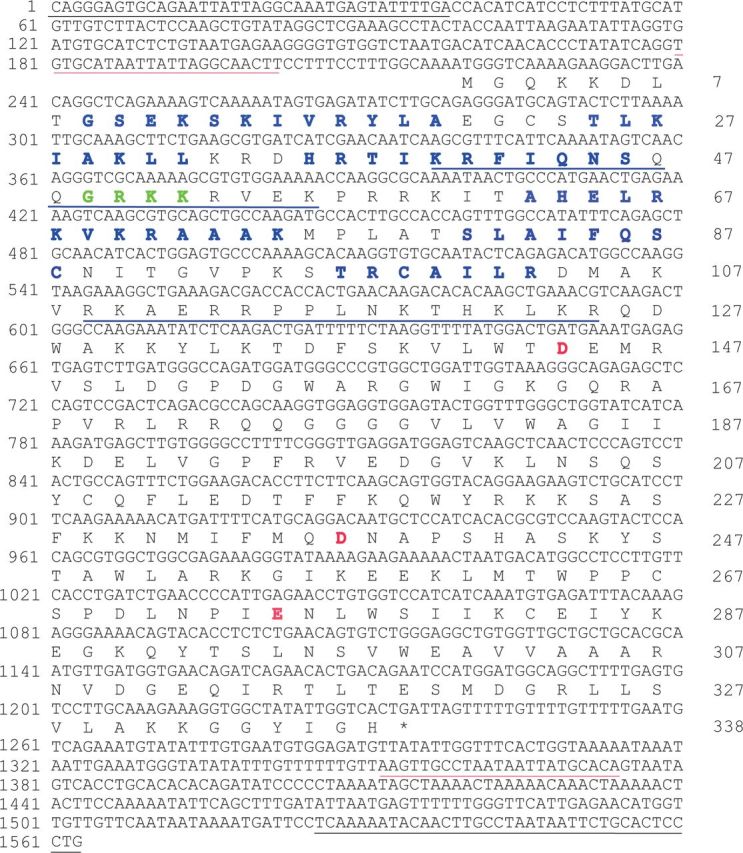
Nucleotide sequence and amino acid translation of *DrTRT*. Its TIRs and SIR are underlined in black and red, respectively. The stop codon is indicated by an asterisk. The six α-helices at the N-terminal of the transposase are indicated by blue letters. A GRKK putative AT-hook like motif is shown by a green letter. Two bipartite NLS at the N-terminal of the transposase are underlined in blue. The catalytic triad DD37E motif within the catalytic domain is indicated by red letters.

Using the deduced protein sequence of *Bmmar1*, we retrieved one sequence (location: CZQB01060324 6427 7413 +) that showed significant similarity (E values: 3e–13) in a TBLASTN search against the zebrafish genome in NCBI. In contrast to the DD37D signature encoded by *Bmmar1*, the spacing of the DDE motif within the catalytic domain of *DrTRT* was DD37E (fig. 1). Although *DrTRT* (named *Mariner-14_DR* in Repbase) has been deposited in Repbase (http://www.girinst.org/repbase/), knowledge and evolutionary history of *DrTRT* remain largely unknown. Therefore, *DrTRT* was utilized in subsequent analyses of *Tc1/mariner* elements in the present study. The nucleotide sequence of *DrTRT* was used as query for BLASTN analysis of the zebrafish genome. All obtained significant hits were extracted with 1,000-bp flanking sequences using our Perl script, and these were aligned to determine their boundaries. The consensus sequence of *DrTRT* was reconstructed, which was determined to be 1,563 bp in length (fig. 1). The transposase was flanked by TIRs that were 38 bp in length (fig. 1). *DrTRT* also contained a 23-bp 5'-subterminal inverted repeat (SIR) (TGTGCATAATTATTAGGCAACTT) that pairs with a reverse complementary 3'-SIR (AAGTTGCCTAATAATTATGCACA) (fig. 1). A previous study suggested that the subterminal region of *DrTRT* plays critical structural or functional roles during transposition (Tu, 2000). *DrTRT* was detected as about 44 copies (table 1). Further analyses showed that 50% (22/44) of *DrTRT* were verified full-length elements that showed >95% sequence identity to the consensus sequence (supplementary table S1, Supplementary Material Online).

**Table 1:** Characteristics of *TRT* and *Tc1* transposons identified in this study

Species	TEs	Length (bp)	TIRs (bp)	Tpase (aa)	Number of copies	Representatives
**Vertebrates**						
**Elopocephala (bony fishes)**						
*Amphilophus citrinellus*	*AcTRT*	1561	27	263	48	emb|CCOE01000287.1|52741355275697+
*Anguilla japonica*	*AnjTRT*	1444	27	217	6	gb|AVPY01162937.1| 2525 3968 –
*Anoplopoma fimbria*	*AnofTRT*	1045	—	—	27	gb|AWGY01162360.1| 2696 3736 -
*Astyanax mexicanus*	*AsmTRT*	1549	28	202	27	gb|APWO01056927.1| 208 1648 +
*Boleophthalmus pectinirostris*	*BpTRT*	1592	44	265	19	gb|JACK01022919.1| 5353 6944 +
*Cynoglossus semilaevis*	*CsTRT*	1552	27	297	150	gb|AGRG01027172.1| 7453 9002 +
*Cyprinodon variegatus*	*CypvTRT*	1570	27	338	254	gb|JPKM01031331.1| 7025 8581 -
*Danio rerio*	*DrTRT*	1563	38	338	44	chr2 13613290 13614851 -
*Dicentrarchus labrax*	*DilTRT*	1282	—	317	12	emb|CABK01017223.1| 2118 3399 -
*Esox lucius*	*ElTRT*	1561	27	323	258	gb|AZJR01046952.1| 21014 22554 +
*Haplochromis burtoni*	*HbTRT*	1563	27	338	252	gb|AFNZ01059715.1| 6228 7790 +
*Larimichthys crocea*	*LcTRT*	1574	30	335	13	gb|JPYK01009954.1| 5998 7571 -
*Maylandia zebra*	*MzTRT*	1563	27	338	251	gb|AGTA02022835.1| 1150 2701 +
*Neolamprologus brichardi*	*NbTRT*	885	—	294	22	gb|AFNY01026505.1| 2222 3106 +
*Nothobranchius furzeri*	*NofTRT*	1011	—	336	6	gb|ABLO01004310.1| 411 1421 +
*Oryzias latipes*	*OlTRT*	1513	28	231	10	dbj|BAAF04056582.1| 7120 8632 +
*Pampus argenteus*	*PaTRT*	982	—	224	46	gb|JHEK01284027.1| 235 1216 +
*Periophthalmus magnuspinnatus*	*PemTRT*	1559	22	338	34	gb|JACL01047866.1| 9655 11168 +
*Pseudopleuronectes yokohamae*	*PsyTRT*	1398	—	250	31	dbj|BBOV01014196.1| 3143 4536 +
*Pundamilian yererei*	*PunTRT*	1563	27	338	257	gb|AFNX01048038.1| 1455 3017 +
*Salmo salar*	*SsTRT*	1561	27	338	435	gb|AGKD04000285.1| 913827 915387 +
*Scartelaos histophorus*	*SchTRT*	1562	27	264	183	gb|JACN01050586.1| 12429 13966 -
*Stegastes partitus*	*StpTRT*	1562	27	338	56	gb|JMKM01022513.1| 24666 26230 +
*Takifugu flavidus*	*TfTRT*	1552	27	338	40	gb|AOOT01008521.1| 14902 16436 +
*Takifugu rubripes*	*TrTRT*	1551	27	338	36	scaffold_12 809695810893 +
*Xiphophorus maculatus*	*XmTRT*	1556	27	250	250	gb|AGAJ01016295.1| 39713 41243 +
**Amphibians (clawed frog)**						
*Xenopus (Silurana) tropicalis*	*XtTRT*	1573	202	326	>45	gb|AAMC02022243.1| 71710 73283 +
**Lepidosauria (snakes)**						
*Crotalus mitchellii pyrrhus*	*CmpTRT*	1562	27	338	216	gb|JPMF01075523.1| 3636 5142 +
*Ophiophagus hannah*	*OhTRT*	1561	26	234	220	gb|AZIM01005391.1| 25631 27415 +
*Python bivittatus*	*PbTRT*	1729	27	320	251	gb|AEQU02140404.1| 2144 3872 +
**Protozoa**						
*Perkinsus marinus*	*PmTRT*	1229	—	289	48	gb|AAXJ01001025.1| 1452 2678 +
**Fungi (Mucorales)**						
*Cokeromyces recurvatus*	*CreTc1*	1672	218	286	60	gb|JNEH01002547.1| 21772 23445 -
*Cunninghamella bertholletiae*	*CbeTc1*	1488	126	343	1	gb|JNEG01000333.1| 1 1488 +
*Lichtheimia corymbifera*	*LcoTc1*	1368	59	303	2	gb|JNEE01000902.1| 112 1479 +
*Mucor circinelloides*	*McTc1*	1579	—	343	53	gb|JNDM01001565.1| 655 2233 –
*Mucor racemosus*	*MrTc1*	1647	38	343	230	gb|JNEI01003901.1| 14238 15884 -
*Mucor ramosissimus*	*MraTc1*	1647	216	242	123	gb|JNEF01002670.1| 804 2448 -
*Mucor velutinosus*	*MvTc1*	1646	33	343	125	gb|JNDK01001598.1| 1060 2704 +
*Rhizopus delemar*	*RdTc1*	1671	211	343	167	gb|AACW02000276.1| 2545 4216 +
*Rhizopus oryzae*	*RoTc1*	1672	211	343	177	gb|JNDV01011981.1| 454 2125 -
*Rhizopus microsporus*	*RmTc1*	861	—	195	24	gb|JNEJ01004884.1| 638 1498 –

The coding domain of the transposase was 1,014 bp (338 amino acids) in length. Several conserved motifs that were characteristics of *Tc1*-like transposons (Plasterk et al. 1999) were also observed in the *DrTRT* sequence (fig. 1). First, there were two helix-turn-helix (HTH) motifs at the N-terminal of the transposase, and each motif consisted of three α-helices. Second, a GRKK putative AT hook-like motif was present between the two HTH motifs. Third, two bipartite NLS were identified at the N-terminal of the transposase. Finally, a catalytic triad DDE motif within their catalytic domain, with 37 amino acids between the second aspartic acid (D) and glutamic acid (E).

### Distribution of *TRT* in Other Sequenced Species

**Fig. 2.—:**
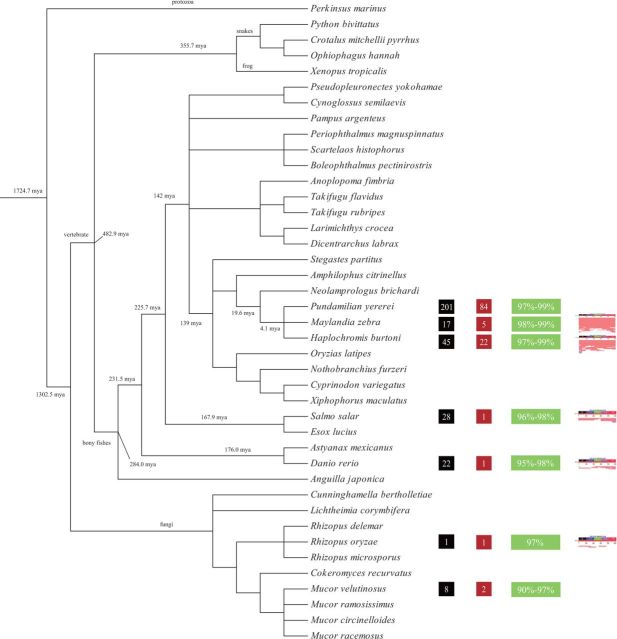
Phylogeny of various species and analysis of potential transposition activity of *TRT* and *Tc1* elements. Numbers in a black box represent numbers of full-length copies in one species. Numbers in a purple box represent numbers of full-length copies encoding intact transposases in one species. Numbers in a green box indicate the sequence identity between full-length copies and consensus sequences in one species. The figures obtained from NCBI show the BLASTN results when searching for transpose nucleotide sequences in the species TSA database.

To determine the species distribution of *TRT*, a TBLASTN search against 1,176 species (437 fungus, 216 invertebrates, 226 vertebrates, 150 plants, and 147 protozoans) whose complete or nearly complete genome sequences are available at NCBI was performed using the protein encoded by *DrTRT* as query. Significant hits encoding the conserved DD37E motifs were detected in bony fishes, clawed frog, snakes, protozoans, and fungi (table 1 and supplementary table S2, Supplementary Material Online). We should notice that *TRT* copies may reside within unassembled portions of a genome because many genomes available in public databases are not truly complete in the sense that they are highly fragmented and transposons are more likely to be interrupted by gaps in the sequence. Their copy number per genome extensively varied among species, changing from 1 copy in the fungi *Cunninghamella bertholletiae* to 435 copies in the Atlantic salmon *Salmo salar* (table 1), thereby suggesting that these transposons underwent species-specific proliferation in their host genomes. Interestingly, the transposases of several full-length copies of *TRT* that were identified in bony fishes and fungi did not harbor internal stop codons or frameshift mutations and presented all expected functional domains, as well as an intact TIR (fig. 2 and supplementary table S1, Supplementary Material Online), suggesting that *TRT* might have current or recent activity in these host genomes. For example, *TRT* identified in the bony fish *P. yererei* had 257 copies, and almost all copies (201/257) were present in full length (table 1 and supplementary table S1, Supplementary Material Online and fig. 2). Meanwhile, all complete copies revealed a strikingly high level of sequence identity (97–99% pairwise nucleotide identity) to the entire length of *TRT* elements, of which 84/201 encoded intact transposases (fig. 2). To further investigate the activity of *TRT*, the nucleotide sequences of the transposases were used as a BLASTN query against the species Transcriptome Shotgun Assembly (TSA) database. Interestingly, when the putative complete transposase sequences were aligned against the full transcriptome database of the bony fishes *M. zebra* and *H. burtoni*, the references were completely covered (100% in length) (fig. 2). Our results also showed that the active *TRT* elements might be restricted to members of Haplochromini (*P. yererei*, *M. zebra*, and *H. burtoni*).The bony fishes *P. yererei*, *M. zebra*, and *H. burtoni* shared the last common ancestor about 4 Ma (Hedges et al. 2006) and they were diverged from the lyretail cichlid *Neolamprologus brichardi* (no active *TRT* was found in this species) about 19.6 Ma, thereby suggesting that the activity of *TRT* elements in these species might have begun at about between 4 and 19 Ma (fig. 2).

**Fig. 3.—:**
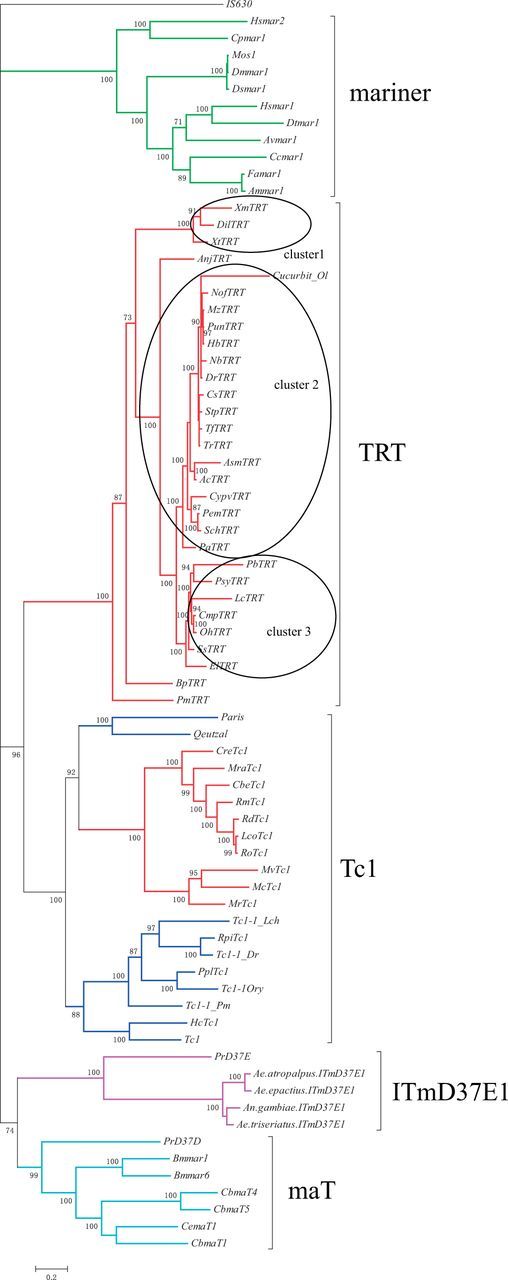
Phylogenetic relationships of all *TRT* and *Tc1* elements identified in this study with other members of the *Tc1/mariner* superfamily based on their transposases. Bootstrap values of <70% are not shown. Three clusters of *TRT* elements might be involved in HTs were circled up in circles. *TRT* and *Tc1* elements identified in this study were shown using red color.

Despite the observation that transposase sequences have consensus sequences with similar lengths, those of TIRs significantly differed between metazoans and fungi (table 1). Generally, *TRT* elements in metazoans except for the clawed frog had a short TIR sequence (26–44 bp) at their termini. *TRT* elements in fungi was flanked by much longer TIRs (generally >100 bp) compared to those of metazoan *TRT* elements. TIRs played important roles (e.g., providing a cleavage signal sequence and binding site for transposases) in the transposition of *Tc1/mariner* transposons (Brillet et al. 2007). *Tc1/mariner* transposons could be divided into different groups based on variations in their TIRs, including length and motif content (Brillet et al. 2007). Therefore, it is important to analyze the functional domain of these length-variable TIRs during transposition by biochemical techniques, as well as compare their structural and functional parts with other transposons of the *Tc1/mariner* superfamily. Phylogenetic analysis of transposases based on their full length indicated that the *TRT* elements of metazoans and fungi could be classified into two independent groups (fig. 3). Interestingly, phylogeny also suggested that *TRT* elements from fungi and reported *Tc1* elements showed much closer relationship when compared with *TRT* elements from metazoans, implying that they might be derived from a more recent ancestor. Indeed, the C-terminal domains of *TRT* elements from fungi and reported *Tc1* elements were very conserved (supplementary fig. S1, Supplementary Material Online). The above results suggest that the *TRT* elements from fungi are members of *Tc1*. Therefore, it is not appropriate to call these elements *TRT*. In order to solve this, all elements from fungi were renamed *Tc1*.

**Fig. 4.—:**
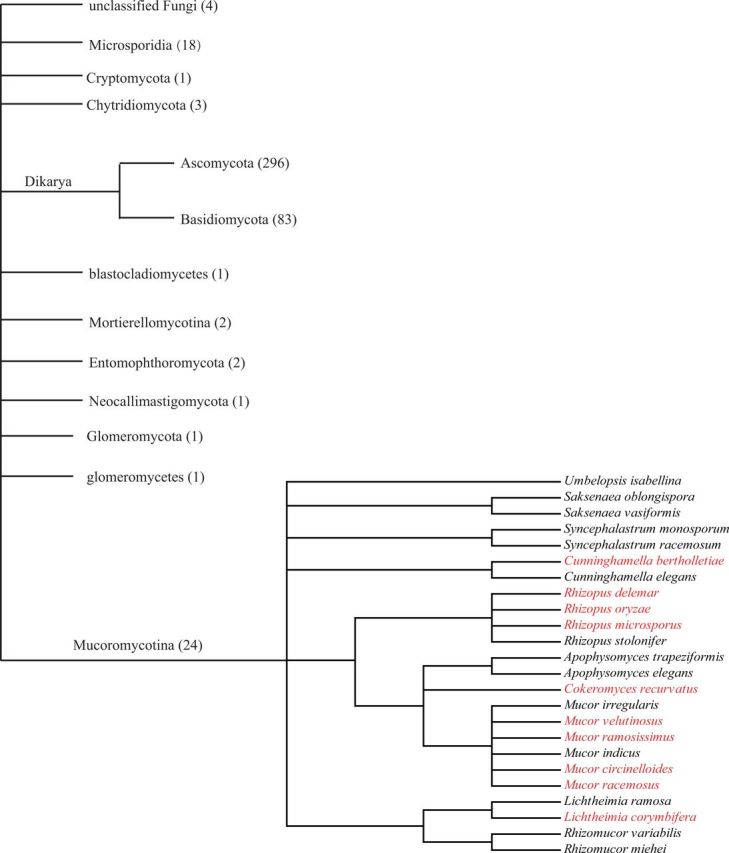
Phylogeny of fungal species with complete or nearly complete genome sequences are available at NCBI. Species harboring *Tc1* elements are shown in red.

Although more than 437 fungal genomes have been sequenced, *Tc1* elements were only detected in fungi belonging to the Mucoromycotina (fig. 4). *Tc1* elements were also extensively distributed in Mucoromycotina, although only 24 the Mucoromycotina species have been completely sequenced. Although the Dikarya embrace two large phyla (the Ascomycota and the Basidiomycota) that account for the vast majority of fungi and most (379/437) of the sequenced fungi, no *Tc1* elements were detected in this taxonomic cluster. We should notice that sampling bias of the available databases may influence our level of detection for *Tc1* elements in fungi. Sequencing of additional fungal genomes may facilitate in the identification of *Tc1* elements. Such expansion will also facilitate in the elucidation of the evolutionary dynamics or patterns of *Tc1* elements in a wide range of fungal species. Several different strains of fungal species have been completely sequenced, and thus we investigated the presence/absence of polymorphisms in the *Tc1* elements of these strains. The fungi *Lichtheimia corymbifera* and *Cunninghamella bertholletiae* harbored polymorphisms in their *TRT* elements (supplementary fig. S2, Supplementary Material Online), thereby suggesting that *Tc1* elements recently invaded their host and currently have not undergone fixation. Alternatively, *Tc1* polymorphisms might result from cut-and-paste excision of the element.

### Comparison of *TRT* Elements with Other Known *Tc1/Mariner* Transposons

To determine the relationship between *TRT* elements and other members of the *Tc1/mariner* superfamily, the transposase sequences of each *TRT* element were aligned to 11 *mariner*, 12 *Tc1*, and 7 *maT* elements. The reported DD37E transposons (*ITmD37E*) were also included in the analysis. The consensus amino acid sequence of the element (*IS630*) from *Shigella sonnei* was used as out-group. The phylogenetic tree showed that *TRT* elements were more closely related to *Tc1* than to *mariner* and *maT* (fig. 3). The bootstrap values obtained using two different methods were all >99%. Our results also showed that *TRT* elements and the reported DD37E transposons formed independent clades, thereby suggesting that these belong to two different subfamilies of the *Tc1/mariner* superfamily.

Next, we compared the functional motifs in the catalytic domain of *Tc1*, *mariner*, *maT*, *ITmD37E*, and *TRT* elements. The results showed that three conserved motifs in the catalytic domain could separate *mariner* (TXDE, HDNA, and SPDLAP(S/T/I)DY) from *Tc1* ((W/F)(S/T)DE, QDND and SPDLNPIE). The three conserved functional motifs of *TRT* elements included (W/F)TDE, Q/HDNA, and SPD/HLNPIE (supplementary fig. S3, Supplementary Material Online), which was similar to those of *Tc1*. However, the *ITmD37E* conserved functional motifs (MDDE, PDLA, and V/CPQA/FRPIE) clearly differed from those of other members of the *Tc1/mariner* superfamily, thereby suggesting that *ITmD37E* could be organized into a distinct family of the *Tc1/mariner* superfamily.

### Evidence for Multiple HTs of *TRT*

**Fig. 5.—:**
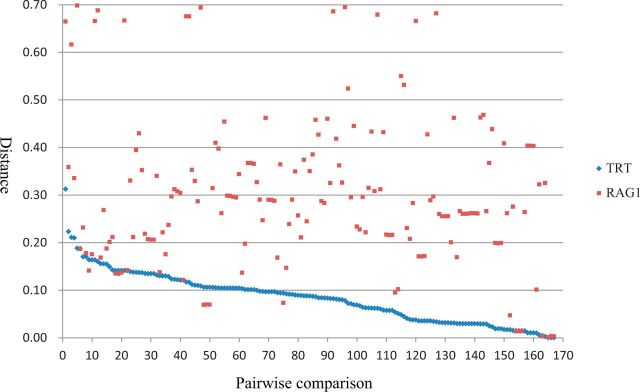
Graph illustrating the pairwise distances of *TRT* and *RAG1* between species included in this study. The distances are obtained from all possible pairwise comparisons (*n* = 167; labeled on the *x* axis) between the 3 (Cluster 1), 17 (Cluster 2), and 8 (Cluster 3) species in which *TRT* was identified.

The above phylogeny showed that *TRT* elements identified in this study could be classified into three major clusters (fig. 3): Cluster 1 includes 3 species (1 frog and 2 bony fishes); Cluster 2 includes 17 bony fishes; Cluster 3 includes 8 species (3 snakes and 5 bony fishes). Phylogenetic analysis also suggested that the host and *TRT* phylogenies were incongruent. This result might imply that *TRT* elements have been exposed to multiple episodes of HTs. To further demonstrate this conclusion, pairwise distances between all consensus sequences were calculated. Distances of almost all pairwise comparisons were extremely low (average = 0.084; SD = 0.053; range = 0.000–0.313) (fig 5 and supplementary table S3, Supplementary Material Online). Meanwhile, most of species involved in *TRT* pairwise distances shared last common ancestor more than 110 Ma (supplementary table S3, Supplementary Material Online). Considering these deep divergence time and the strikingly low-pairwise *TRT* distances seem incompatible with the possibility that these transposons were obtained from parents to offspring. Indeed, for almost all pairwise comparisons (153/167), the distances computed for *TRT* are much lower than those calculated for *RAG1* (average = 0.302; SD = 0.160; range = 0.001–0.698) (supplementary table S3, Supplementary Material Online), which was usually used to infer the HT events of transposons in vertebrates (Gilbert et al. 2010; Gilbert et al. 2012). Two additional lines of evidence ruled out the scenario that *TRT* elements were vertically inherited from the last common ancestor of these species. First, *TRT* elements identified in closely related bony fishes showed a higher level of nucleotide sequence divergence than those of bony fishes and snakes (data not shown). Second, the taxonomic distribution of *TRT* elements was highly discontinuous. Although more than 226 vertebrates have been sequenced, *TRT* elements were only detected in bony fishes, frog, and snakes. Together, our results showed that the presence of *TRT* in most of species reported in this study is as a result of HT events.

However, for 14 pairs of species, we also noticed that the distance computed between *TRT* was greater than the distances calculated for *RAG1* (supplementary table S3, Supplementary Material Online). Two hypotheses may explain this phenomenon: First, *TRT* was transferred into the common ancestor of comparison species by HTs and these species got *TRT* by vertical transfers; Second, *TRT* was introduced in each species by HTs but the happening time of these HT events and the species diverged was very close. This phenomenon was also observed in other vertebrates (Gilbert et al. 2012).

In conclusion, our findings indicate that vertical and HTs may not be mutually exclusive and may have concurred in the evolution of *TRT* elements.

## Discussion

### Description of a *Tc1*-like Transposon

The present study identified a *Tc1*-like transposon in zebrafish. *DrTRT* presented all the hallmark features of *Tc1*-like elements, including the existence of a transposase of about 340 amino acids in length, a DDE motif, two HTH motifs in the DNA-binding domains, TIRs, and TSD TA at each end. The spacing of the DDE motif within the catalytic domain of *DrTRT* was unique, with 37 amino acids (DD37E) separating the second aspartic acid and the glutamic residues. Although *ITmD37E* has been previously reported (Shao and Tu 2001), phylogenetic analysis and functional domain comparison demonstrated that *TRT* elements and *ITmD37E* belong to two distinct subfamilies of the *Tc1/mariner* superfamily. Phylogenetic analysis also showed that *TRT* elements belong to the *Tc1* family (fig. 3). However, the observed high genetic distance with the rest of the *Tc1* elements (DD34/35E) and the presence of a unique DDE spacing (DD37E) suggest that *TRT* elements constitute a novel subfamily within the *Tc1* family.

The present study observed different species distributions for *TRT* and *ITmD37E* elements. *TRT* elements were detected in the genomes of bony fishes, clawed frog, snakes and protozoans following a search in GenBank. No *TRT* elements were observed in insects. In contrast, previous studies and our results (data not shown) indicated that *ITmD37E* extensively occurred in various species, including Arthropoda, Rotifera, flatworms, Hydrozoa, and ciliates (Shao and Tu 2001; Arkhipova and Meselson 2005; Biedler et al. 2007). On the other hand, *ITmD37E* was not detected in vertebrates.

### *TRT* Elements Are Complete and Potentially Active in Haplochromini

To date, only some *Tc1*-like elements (such as *Tol1*, *Tol2*, *Passport*, and *Tana1*) that were identified in bony fishes have been demonstrated to be transpositionally active (Koga et al. 2006; Clark et al. 2009; Pujolar et al. 2013; Watanabe et al. 2014). Previous studies showed that most *Tc1*-like elements in fishes are inactive because of stop codons, deletions, or frameshifts within their sequences (Radice et al. 1994; Reed 1999). In contrast, *TRT* elements were determined to be intact in the genomes of *P. yererei*, *M. zebra*, and *H. burtoni*. These transposases showed no internal stop codons or frameshift mutations, and their TIRs were completely identical, thereby suggesting that *TRT* elements have current or recent activity in these species. Generally, inactive transposons are expected to rapidly accumulate random mutations not only in the transposase but also in the TIRs, thereby disrupting their complementarity (Pujolar et al. 2013). Recent activity of *TRT* elements is also supported by the observations of a very high sequence identity that was observed between full-length copies and consensus sequences, high copy number, and the presence of several elements in the TSA databases (fig. 2). Although intact transposases of *TRT* or *Tc1* elements were also identified in other species, including *Salmo salar*, *D. rerio*, and fungi, no complete transposases was detected in their transcriptomes (fig. 2). These findings suggest that *TRT* elements might not be active in these species. Further analysis suggested that the activity of *TRT* elements in *P. yererei*, *M. zebra*, and *H. burtoni* might have begun about between 4 and 19 Ma.

Homology-based analysis indicates that *TRT* elements may have occur as approximately 250 copies in *P. yererei*, *M. zebra*, and *H. burtoni* (table 1). Similar copy numbers were also observed for the other two active *Tc1*-like elements, *Passport* (300 copies) and *Tana1* (764–1,568 copies) (Leaver 2001; Pujolar et al. 2013). Furthermore, the detection of *Passport* and *Tana1* in divergent species also suggests that HT played important roles in their dissemination (Leaver 2001; Pujolar et al. 2013).

### Mechanisms and Direction of HT Events Involving *TRT* Elements

Although we have demonstrated that *TRT* elements underwent HT events to bony fishes, snakes and frogs, their underlying mechanisms remain unknown. However, it should be noted that DNA viruses are well recognized as potential intermediates for HT of transposons among various animal species (Schaack et al. 2010). Interestingly, it is reported that a snake retroposon integrated into the genome of the taterapox virus, a poxvirus that infects West African rodents, by HT (Piskurek and Okada 2007). Therefore, we speculate that poxviruses may be an efficient vector for the HT of *TRT* elements that were identified in snakes. Parasitism might also facilitate the HT of transposons. Recently, some cases of HT events involving transposons between vertebrate and their parasites were reported (Kuraku et al. 2012; Walsh et al. 2013; Zhang et al. 2014; Suh et al. 2016). In the present study, a high sequence identity of *TRT* elements was also observed in several bony fishes, indicating that parasitism might have served as an effective delivery system for the HT of *TRT* elements.

A second important issue that needs to be addressed is the direction of HT. In the present study, the continuous distribution of *TRT* elements across bony fish genomes and *TRT* elements from snakes and frog are nested within the bony fishes (fig. 3), thereby suggesting HT events from the bony fishes to the snakes or frog.

### Potential Applications

The discovery of a widespread *Tc1*-like transposon in organisms may have potentially important applications. Molecular tools are being developed for the genetic manipulation of organisms. Active *Tc1/mariner* transposons could be potentially used as molecular tools for transgenesis and insertion mutagenesis (Pavlopoulos et al. 2007; Voigt et al. 2016). The integrity of the native form (with an intact transposase), the presence of all functional domains, and their occurrence in the *M. zebra* and *H. burtoni* transcriptomes are suggestive of current or recent activity of *TRT* elements. Meanwhile, *TRT* elements are widespread in organisms. Therefore, we speculate that *TRT* elements identified in this study might be used as a molecular tool.

## Supplementary Material

Supplementary Data
