# Supplementary material for: TRT, a Vertebrate and Protozoan Tc1-Like Transposon: Current Activity and Horizontal Transfer
Source: Genome Biol Evol. 2016 Sep 25;8(9):2994–3005. doi: 10.1093/gbe/evw213 (PMC5630946; doi:10.1093/gbe/evw213)
Supplement: Supplementary Data [file evw213_Supplementary_Data.zip › supplementary_Figure_S3.pdf]

| Group | Sequence | | Alignment |
|---|---|---|---|
| ITmD37E1 | PrD37E | : | MDDEKYFKLSGDNVLGNRYFYSNDPSTAPPDVKFQKKAKFERKVMVWIAISSRGISSVYVHKSKQAVRQ--ETYLAECIDKRLL-----PFIEKHHS--DG-KYLFWPDLATSHYSNIVQQRLRD-NHIPYVLRIDNPPNVPQARPIE- |
| | An.gambiae.ITmD37E1 | : | MDDETYVKADFGQIPGQTFYLATGRGDVPAKFKFVFADKFARKFMIWQGICSCGKKTKVFVTN-KTMTS--ELYQKECLQKRIL-----PFIRSHDH-----PVMFWPDLASCHYSKVVREWYAE-KGVLFVPKNLNPPNCPQFRPIE- |
| | Ae.atropalpus.ITmD37E1 | : | MDDETYVKAEFGQIPGQKIYLATARGDVSAKFKFVFADKFARKNIIWQGICSCGQKTKVFLTD-KTMTS--EVYKKECLQKRIL-----PFIRAHDR-----PVMLWPDLASCHYSKTVIEWYAT-NGVSVIPKDLNPPNCPQFRPIE- |
| mariner | Mos1 | : | TGDEKWIFFVNPKRKK---SYVDPGQP-ATSTARPNRFGKKTMLCVWWD--QSGVIYYELLKPGETVNT--ARYQCQLINLNRALQRKRPEYQKRQH-----RVIFLHDNAPSHTARAVRDTLET-LNW---EVLPHAAYSPDLAPSDY |
| | Hsmar1 | : | TCDEKWILYDNRRRPA---QWLDREEA-PKHFPKPNLHQKKVMVTVWWS--AAGLIHYSFLNPGETITS--EKYAQQIDEMHRKLQ---RLQPALVN--RK-GPILLHDNARPHVAQPTLQKLNE-LGY---EVLPHPPYSPDLSPTDY |
| | Famar1 | : | TGDEKWVVYNNIKRKR---SWSRPGEP-AQTTSKAGIHQKKVLLSVWWD--YKGIVYFELLPPNRTINS--VVYIEQLTKLNNAVE---EKRAELTN--RK-GVVFHHDNARPHTSLVTRQKLLE-LGW---DVLPHPPYSPDLAPSDY |
| | Ammar1 | : | TGDEKWVVYNNIKRKR---SWSRPREP-AQTTSKAGIHRKKVLLSVWWD--YKGIVYFELLPPNRTINS--VVYIEQLTKLNNAVE---EKRPELTN--RK-GVVFHHDNARPHTSLVTRQKLLE-LGW---DVLPHPPYSPDLAPSDY |
| maT | PrD37D | : | FTDEKYFSLEGVFNRQNERVYAVSRDDADQQGGINQTSKYPKRIMIWLGASENGLTSPIIFQPG-ETLH--ENYINVVLPHARA-------EGERLL--GT-DFIYQQDNATPHVHRKSLTWCAE-NFSNFIDNERWPPNSPDLNVLDY |
| | Bmmar1 | : | FTDDKIFTIEQHFNKQNDRIYAQSSKEASQLVDRVQRGHYPTSVMVWWGVSYEGVTGPYFCEKGIKTLA--QVYQGTILEKVVK-----PLNNTMFN--NQ-EWSFQQDSAPGRKARSTQSWLET-KVLDCIRAEDWPSSSPDLNPLDY |
| | CemaT1 | : | FTDEKIFCIEQSFNTQNDRVYAK-----TQPNSRVQRTGYPKGIMVFAGITANGKTPLIFVPQGIKVNG--NNYLDMLKTELM------PWVKKHFK--KT-KWTFQQDGAPAHKHKNVQAWYES-NFPDFIAFNQWPPSSPDLNPMDY |
| | CbmaT1 | : | FSDEKLFSVEAEFNSQNHRVLATNIQQAAERGKTIHRASHPASIMVFGAVCYDGKCPLVFVDKGVKINQ--KYYVEEILEKHVL-----PWAQNHFG--QK-KYIFQQDGAPAHTAKMAQQWCKD-HFPAFIPKDEWPASSPDLNPLDY |
| TRT | DrTRT | : | WTDEMRVSLDGPDGWA--RGWIGKGQRAPVRL---RRQQGGGGVLVWAGIIKDELVGPFRVEDGVKLNS--QSYCQFLEDTFFK-----QWYRKKSASFKK-NMIFMQDNAPSHASKYSTAWLAR-KGIKEEKLMTWPPCSPDLNPIE- |
| | XtTRT | : | FTDECRATLDGPDGWS--SGWLVEGHRLPTRL---QRQQGGGGVMFWAGIMGRELVGPFRVPEGVKMTS--AKYVEFLTDHFL------PWYRRKNYAFRN-KIIFMHDNAPSHAAKNTSASMAA-MGIKGEKVMVWPPSSPHLNPIE- |
| Tc1 | Impala | : | WSDECMVRRGQGMRPI--WTFLSPREALRVQDVQEARRLGAVRQMFWAAFGHRSRTPLVPLVGNVNAIGIYELYSFIL-----------PWFLQS-------GDIFMHDNASVHTARIVKALLEE-LGV---DLMTWPPYSPDLNPIE- |
| | Bari-1 | : | WTDESAFQYQGSYSKH----FMHLKNNQKHLAAQPTNRFGGGTVMFWGCLSYYGFGDLVPIEG--TLNQ--NGYLLILNNHAF------TSGNRLFP--TT-EWILQQDNAPCHKGRIPTKFLND-LNL---AVLPWPPQSPDLNIIE- |
| | Paris | : | FCDETKIMLYYHDGPS--KVWRKPNTALEQKNIIPTVKFGKLSVMVWGCISSKGVGELRIFND--VMTK--EFYLDILKNELSRSAIKFGFVDPQNP--SKQRYKLYQDNDPKHKSFLCRTWLLY-NCS---KVIDTPAQSPDLNPIE- |
| | Qeutzal | : | FTDESKFNIFGWDGTI--KVWRPPGEGLNPKYTAKTVKHNGGGVLVWGCMAANGVGNLQVIDG--IMDQ--YVYINILKQNLG------PSLEKLGM--SQ-DYWFQQDNDPKHTAFNSRLFLLY-NTP---HQLKSPPQSPDLNPIE- |
| | Tc1 | : | WSDESKFNLFGSDGNS--WVRRPVGSRYSPKYQCPTVKHGGGSVMVWGCFTSTSMGPLRRIQS--IMDR--FQYENIFETTMR------PWALQNV---GR-GFVFQQDNDPKHTSLHVRSWFQR-RHV---HLLDWPSQSPDLNPIE- |
