# Supplementary figures and images for: TRT, a Vertebrate and Protozoan Tc1-Like Transposon: Current Activity and Horizontal Transfer
Source: Genome Biol Evol. 2016 Sep 25;8(9):2994–3005. doi: 10.1093/gbe/evw213 (PMC5630946; doi:10.1093/gbe/evw213)

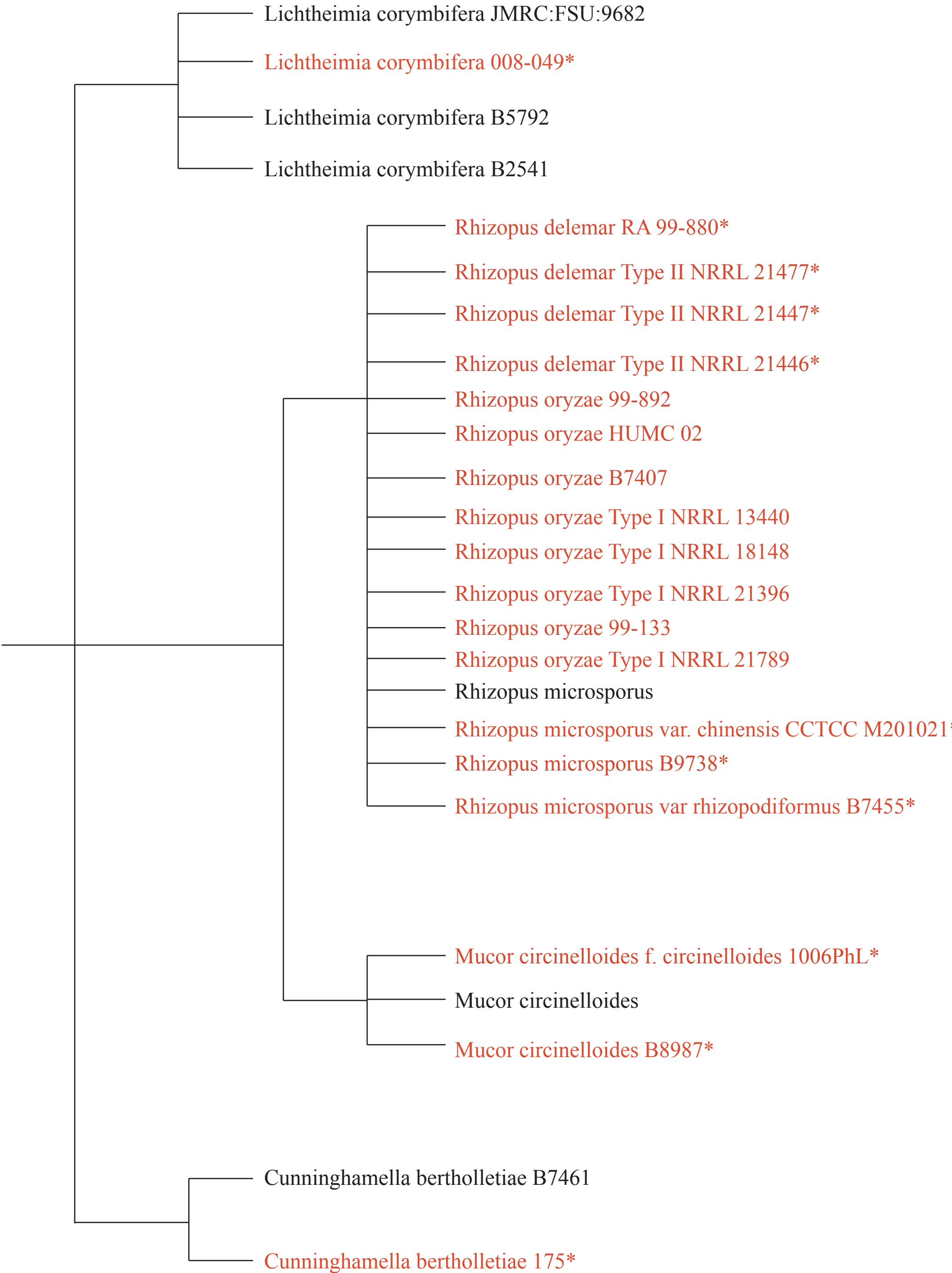

Supplement: Supplementary Data [file evw213_Supplementary_Data.zip › supplementary_Figure_S2.pdf]
