# Supplementary material for: TRT, a Vertebrate and Protozoan Tc1-Like Transposon: Current Activity and Horizontal Transfer
Source: Genome Biol Evol. 2016 Sep 25;8(9):2994–3005. doi: 10.1093/gbe/evw213 (PMC5630946; doi:10.1093/gbe/evw213)
Supplement: Supplementary Data [file supplementary_Figure_S1.pdf › supplementary_Figure_S1.pdf]

Tc1

```
Tc1-1_Lch   : QHDNDPKHTAKSTLEFLKRRKI---KVLEWPSQSPDLNPIE-MLWSDLKKAVA--KHCVSNLSELKEICKTEWANIPPER-CKTLVRNYHKRLTAVIKAKGGH-TKY--
RpiTc1      : QQDNDPKHTSKSTTEWLKKNKM---KTLEWPSQSPDLNPIE-MLWYDLKKAVH--ARKPSNVTELGQFCKDEWAKIPPGR-CKSLIARYRKRLVAVVAAKGGP-TSY--
Tc1-1_DR    : QQDNDPKHTSKSTSEWLKKNKM---KTLEWPSQSPDLNPIE-MLWHDLKKTVH--AQKPSNVAELQQFCKDEWAKIPPQR-CNRLIASYRKCLIAVVAAKGGP-TSY--
Tc1-1_PM    : QHDNDPKHTAKATKEWLKKKHI---KVLEWPSQSPDLNPIE-NLWRELKVRVA--KRQPRNLNDLEKICKEEWDKIPPEM-CANLVANYKKRLTSVIANKGFA-TKY--
PplTc1      : QQDNDPKHTARATKEWF---GLKNVNVLKWPSQSPDLNPIE-NLWQDLKIAVH--RRSPSNLTELHLFCQEEWTNLSISR-CAKLVETYPKRLAAVIAAKGGS-TKY--
Tc1-1Ory    : QXNNDPKHTAKSTMDWFTNKHI---QVLEWPSQSQDLNRIE-NLWKELKTAVH--KHSPSNLTELELXCKEEWAKMSVSQ-CEKLIETYSKRFTAVITAKGGG-TK---
Qeutzal     : QQDNDPKHTAFNSRLFLLYNTP---HQLKSPPQSPDLNPIE-HAWELLERKIR--QTRIKNRVDLENKLKEAWITISEDY-TQNLVNSMPRRLAEVIKMKGYA-TRY--
Paris       : YQDNDPKHKSFLCRTWLLYNCS---KVIDTPAQSPDLNPIE-NLWAFLKKRVG--KRSPTNKNALIKAIQEEWIKIPEIYDLHNLIQSMSRRLRAVMDANGQY-TKY--
HcTc1       : QQDNDPKHKSKLLTKWFRDNNV---PLLMWPSLSPDLNATE-NLWERLKHQVK--GLRARNEHEKFNQLKTAWENIPQEE-IDKLIESMPCRCQAVIDARGHA-TKY--
Tc1         : QQDNDPKHTSLHVRSWFQRRHV---HLLDWPSQSPDLNPIE-HLWEELERRLG--GIRASNADAKFNQLENAWKAIPMSV-IHKLIDSMPRRCQAVIDANGYA-TKY--
```

```
ReTc1       : QQDKDPKHTAKLTKQWLADNKFDIESTYSWPPQSPDLNPIE-HIWHHLKLKLSAYPTKAKGVHELWERIEEQWATFTKED-CQKYIDSMPARIKAVIDAKGGY-TKY--
```

Tc1 from fungi

```
MraTc1      : QQDNDPKHTSKLAHAWFKENGFKEEHTFHWPAQSPHLDPIE-HLWHHLKLRLSLYEGRAKGVHDLWSRIEEEWNSFTKEQ-CQAYIKSMPARCRAVIKAKGGY-TRY--
CbeTc1      : QQDNDPKHTSKLAKQWFKDNNFKCDHTFNWPSQSPDLNPIE-HIWHHLKLKLSAYDTRAKGVHELWNRVEKEWSTFTAED-CRRYINSMPDRCRAVIAAKGGH-TRY--
RmTc1       : QQDNDPKHTSKLAKEWFASNGFKTDHIFNWPAQSPNLNPIE-HVWHHLKLKLSAYETKPKGVHELWDRIEKEWNTFTAEE-CRRYVNSMLDRYRAVIDANGGH------
RdTc1       : QQDKDPKHTSKLAKQWFVDNNFNSDHIYSWPAQIPDLNPIE-HVWHHLKLKLSVYDTKAKGVHELWERIEKEWNTFTVDE-CRRYIDSMPDRCKAVIKAKGGH-TRY--
LcoTc1      : QQDKDPKHTSKLAKQWFVDNNFKSDHIFSWPAQSPDLNPIE-HVWHHLKLKLSVYDTKAKGVHEL--------------------------------------------
RoTc1       : QQDKDPKHTSKLAKQWFVDNNFKSDHIFSWPAQSPDLNPIE-HVWHHLKLKLSVYDTKAKGVHELWERIEKEWNTFTVDE-CRRYIDSMPDRCKAVINAKGGH-TRY--
MvTc1       : QQDRATPHTSGITKQWFTDNGFNLDEIMDWPPQSPDLNPIE-HVWHLLKLRLNKYPTRPSTKEELERRINIQWYKITEKE-CQKYIDSMPARIKAVIKSKGGS-THY--
MrTc1       : QQDNATPHTSVPTKQWFQRQGFSLKSILDWPPQSPDLNPIE-HVWNQLKRRLNAYPARATTIAELEARIHQEWYKFTKED-CLKYIDSMPNRIKAVIRSKGGP-TRY--
McTc1       : QQDKASPHKSVVTKKWCTDNGFSADEILDWPAQSPDLNPIE-HVWSELKRRLDTYKRCPTTKEELANRISTEWDKFTKED-CLRYIYSMPKRIEAVIKSNGGP-TTY--
```

TRT from metazoans

```
XmTRT       : MHDNAPSHAARNTTVSLAAMGIKGEKLMVWSPSSSDLNPIE-NLWSFLKQKI---------------------------------------------------------
XtTRT       : MHDNAPSHAAKNTSASMAAMGIKGEKVMVWPPSSPHLNPIE-NLWSILKQKIYEGGRQFTSKQQLWEAILTSCKQIQAET-VQKLTSSMDARLVKLLSNKGSY-VKM--
DilTRT      : IHDNAPSHAAKNTSVSLAAMGIKGEKLMVWPPSSPDLNPIE-NLGRILKQKIYEGGRQFTSKQQLWEAVLTSCKDIQAET-LQQLTSSMDARIVKAISKKGSY-VNM--
BpTRT       : MHDNAPSHASKFTAEWLASKGYLQDKIMDWPASSPDLNCIE-NLWLILKRRVFENGKQFSSKDKLWEAIVKAAKSIKPAE-IENLTSSMDNRLLTLMQKKGGY-ISK--
PmTRT       : MQDNAPSHASKFTKDYLEKKGFTGRKLMNWPPNSPDINPIE-RYWSILKK-----------------------------------------------------------
AnjTRT      : MQDNAPSHASQYTRDWLASKGFKDGKMMTWPPCSPDLNPIE-NLWALLKREIYSQGKQYTSLNSLWASLLAASAKIGRDP-IKKLTESVDMRLIQVLEKKG--------
OlTRT       : MQDDAQSHASKYFTAWLARKGLKEEKLMTWPPCSPDLNPIE-NLWSIMKCEIYKEGKQYFS------------------------------------------------
PbTRT       : MQDNAPSHASKYSTAWLASNGLKDERIMTWPPSTPDLNPTE-NLWALLKREIYGEGKQYTPLNSVWEAAQKVE-----CQ-IKKLTDAMNGRLMTVIAKKGGY-IGH--
AsmTRT      : MQDNAPSHASKYSTAWLASKGLKEEKIMTWPPCSPDLNPIE-NLWSLIKCEIYREGKQYTSLNSVWEAVVAAARNVDRKQ-IKKLTESMDGRLLSVIVKKGGY-IGH--
ElTRT       : MQDNAPSHASKYSTAWLASKGLKDARIMTWPPSLPDLNPIE-NLWTLLKREIYSQGRQYTSLNSIWEAVVAASAKVDREQ-IKKLTDCMDGRLMAVIEKKGGY-IGH--
CypvTRT     : MQDNAPSHASKYTTAWLARKSLKGEKIMTWPPCSPDLNPME-NLWSFMKCEIYREGKQSTSLNSVWEAVVAAARNVDRKQ-IKTLTESMDGRLLSVMVKKGGY-IGH--
PaTRT       : MQDNAPSHASKYSTAWLASKGLKGEKIMTWPPCSPDLNPIE-NLWSIIKREVYKEGKQYTSLNSVWEAVVAAAHNVGREQ-IKKLTESMDGRLLSVLMKK---------
PemTRT      : MQDNAPSHASKYSTAWLASKGLKDEKIMTWPPCSPDLNPIE-NLWSLMKRKIYKEGKQYTSLNSVWEAVVAAARNVDGEQ-IKKLTESMDGRLLSVLVKKGGY-IGH--
SchTRT      : MQDNAPSHASKYSTAWLASKGLKDEKIMTWPPCSPDLKPIE-NLWSLMKRKIYKEGKQYTSLNSVWEAVVAAARNVDGEQ-IKKLTESMDGRLLSVLVKKGGY-IGH--
AcTRT       : MQDNAPSHASKYSTAWLASKGLKEEKIMTWPPCSPDLNPIE-NLWSLIKCEIYREGKQYTSLNSVWEAVVAAARNVDRKQ-IKQLTESMDGRLLSVIVKKGGY-IGH--
NofTRT      : MQDNAPSHMSKYSTAWLARKGIKEEKLMTWPPCSPDRNSIE-NLWSIIKCEIYKEGKQYTSLNSVWEAVVAAARNVDGEQ-IKTLTESMDGRLLSVLAKKGGY-IG---
NbTRT       : MQDNAPSHASKYSTAWLARKGIKEEKLMTWPPCLPDLNPIE-NLWSIIKCEIYKEGKQYTSLNSVWEAVVAAARNVDGEQ-ITTLTESMDGRLLRV-------------
DrTRT       : MQDNAPSHASKYSTAWLARKGIKEEKLMTWPPCSPDLNPIE-NLWSIIKCEIYKEGKQYTSLNSVWEAVVAAARNVDGEQ-IRTLTESMDGRLLSVLAKKGGY-IGH--
MzTRT       : MQDNAPSHASKYSTAWLARKGIKEEKLMTWPPCSPDLNPIE-NLWSIIKCEIYKEGKQYTSLNSVWEAVVAAARNVDGEQ-IKTLTESMDGRLLSVLAKKGGY-IGR--
PunTRT      : MQDNAPSHASKYSTAWLARKGIKEEKLMTWPPCSPDLNPIE-NLWSIIKCEIYKEGKQYTSLNSVWEAVVAAARNVDGEQ-IKTLTESMDGRLLSVLAKKGGY-IGR--
HbTRT       : MQDNAPSHASKYSTAWLARKGIKEEKLMTWPPCSPDLNPIE-NLWSIIKCEIYKEGKQYTSLNSVWEAVVAAARNVDGEQ-IKTLTESMDGRLLSVLAKKGGY-IGR--
CsTRT       : MQDNAPSHASKYSTAWLARKGLKEEKLMTWPPCSPDLNPIE-NLWSLIKCEIYKEGKQYTSLNSVWEAVVAAARNVDREQ-IKTLTESMDGRLLSVLAKKGGY-IGH--
StpTRT      : MQDNAPSHASKYSTAWLARKGLKEEKLMTWPPCSPDLNPIE-NLWSLIKCQIYKEGKQYTSLNRVWEAVVAAARNVDREQ-IKTLTESMDGRLLSVLAKKGGY-IGH--
TfTRT       : MQDNAPSHASKYSTAWLARKGLKEEKLMTWPPCSPDLNPTE-NLWSLIKCEIYKEGKQSTSLNSVWEAVVAAARNVDREQ-IKTLTESMDGRLLSVLAKKGGY-IGH--
TrTRT       : MQDNAPSHASKYSTAWLARKGLKEEKLMTWPPCSPDLNPIE-NLWSLIKCEIYKEGKQYTSLNSVWEAVVAAARNVDREQ-IKTLTESMDGRLLSVLAKKGGY-IGH--
PsyTRT      : MQDNAPLHASKYSTVWLASKGLKDEKIMTWSPSSPDLNTIE-NLWALFKREIYSEGKQYTSLNSVWEAVDAAAQNVDRQQ-IKKLTESMNGRL----------------
SsTRT       : MQDNAPSHASKYSTAWLASKGLKDERIMTWPPSSPDLNPIE-NLWSLLKRTIYGEGKQYTSLTSVWEAVVAAAQKVDGQQ-IKKLTDSMDGRLMTVIEKKGGY-IGH--
CmpTRT      : MQDNAPSHASKYSTAWLASKGLKDERIMTWPPSSPDLNPIE-NLWALLKWEIYGEGKQYTSLNSVWEAVVAAAQNVDCQQ-IKKLTDSMNGRLMTVIEKKGGY-IGH--
OhTRT       : MQDNAPSHASKYSTVWLASKGLKDERIMTWPPSSPDLNPIE-NLWALLKWEIYGEGKQYTSLNSVWEAVVAAAQKVDRQQ-IKKLTDSMNGRLMTVIEKKGGY-IGH--
```

D34/37E
